# An Information-Entropy Position-Weighted *K*-Mer Relative Measure for Whole Genome Phylogeny Reconstruction

**DOI:** 10.3389/fgene.2021.766496

**Published:** 2021-10-22

**Authors:** Yao-Qun Wu, Zu-Guo Yu, Run-Bin Tang, Guo-Sheng Han, Vo V. Anh

**Affiliations:** ^1^ Hunan Key Laboratory for Computation and Simulation in Science and Engineering and Key Laboratory of Intelligent Computing and Information Processing of Ministry of Education, Xiangtan University, Hunan, China; ^2^ Provincial Key Laboratory of Informational Service for Rural Area of Southwestern Hunan, Shaoyang University, Shaoyang, China; ^3^ Faculty of Science, Engineering and Technology, Swinburne University of Technology, Hawthorn, VIC, Australia

**Keywords:** alignment-free method, k-mer relative distance, information entropy, phylogenetic analysis, genome

## Abstract

Alignment methods have faced disadvantages in sequence comparison and phylogeny reconstruction due to their high computational costs in handling time and space complexity. On the other hand, alignment-free methods incur low computational costs and have recently gained popularity in the field of bioinformatics. Here we propose a new alignment-free method for phylogenetic tree reconstruction based on whole genome sequences. A key component is a measure called *information-entropy position-weighted k-mer relative measure* (IEPWRMkmer), which combines the position-weighted measure of *k*-mers proposed by our group and the information entropy of frequency of *k*-mers. The Manhattan distance is used to calculate the pairwise distance between species. Finally, we use the Neighbor-Joining method to construct the phylogenetic tree. To evaluate the performance of this method, we perform phylogenetic analysis on two datasets used by other researchers. The results demonstrate that the *IEPWRMkmer* method is efficient and reliable. The source codes of our method are provided at https://github.com/ wuyaoqun37/IEPWRMkmer.

## Introduction

The reconstruction of a phylogenetic tree is a primary problem in evolutionary biology. Sequence alignment is a key step in the reconstruction, aiming to identify the homology of sequences and uncover phylogenetic relationships in sequences. Traditional sequence comparison is based on pairwise or multiple sequence alignment (Felsenstein and Felenstein, 2004; Morrison, 2006) and was implemented by software packages such as BLAST (Altschul et al., 1990), ClustalW (Thompson et al., 1994), and MrBayes (Ronquist et al., 2012). However, the methods based on sequence alignment have some disadvantages, including high computational cost in handling the time and space complexity of the algorithm. Therefore, alignment-free methods have been proposed to overcome these problems (Zielezinski et al., 2017). The computational cost of alignment-free methods is low because they are generally of linear complexity (Fox et al*.*, 1977).

Several alignment-free methods for sequence comparison are based on word counts (Blaisdell, 1986; Höhl et al., 2006; Wang et al., 2016). A key idea is to use the close distribution of *k*-mers to imply the high correlation degree, hence the similarity of the sequences. The methods have been implemented in software tools, such as FFP (Sims et al., 2009), kWIP (Murray et al., 2017), CVtree (Qi et al., 2004), and DLtree (Wu et al., 2017). Many *k*-mer methods transform the input sequence into a frequency vector of *k*-mers, then define the distance of the sequences by that of the frequency vector of *k*-mers (Qi et al., 2004; Wu et al., 2017). To reduce the statistical dependence between adjacent word matches, Spaced-Words (Leimeister and Boden, 2014) proposed to use spaced words, which are defined by patterns of matches without reference to positions. Some alignment-free methods are based on match length, which defines the distance between sequences based on the length of substring matches between two sequences. These include the shortest unique substring method (Haubold et al., 2005), ACS (Ulitsky et al*.* 2006), UA (Comin and Verzotto, 2012), and ALFRED (Thankachan et al*.* 2016). In addition, graphical representation was used to construct the probability distribution of a DNA sequence (Yu et al., 2011). The chaos game representation transforms the distribution of characters in a DNA sequence into the distribution of nodes in a graph (Hoang et al*.* 2016; Yin, 2017; Mendizabal-Ruiz et al., 2018). Many researchers considered extracting the position information of a *k*-mer (Huang and Wang, 2011; Ding et al., 2013; Tang et al., 2014). Ding et al. (2013) used the average interval distance of normalized *k*-mers to capture evolutionary information for sequence comparison. Tang et al. (2014) presented the average relative distance of normalized *k*-mers to improve the method of Ding et al. (2013). Ma et al. (2020) proposed the *PWKmer* method, which combines the *k*-mer counts and *k*-mer position distributions for phylogenetic analysis.

In this work, we propose a new alignment-free method which combines the position-weighted measure of *k*-mers proposed by Ma et al. (2020) and the information entropy of frequency of *k*-mers to obtain phylogenetic information for sequence comparison. It is named *information-entropy position-weighted k-mer relative measure* (IEPWRMkmer). To evaluate the performance of this method, we carry out phylogenetic analysis on two data sets used by other researchers.

## Materials and Methods

### Genomic Datasets

#### Dataset 1

The first dataset for analysis consists of the same whole genome DNA sequences of 30 mammalian species studied in Li et al. (2001), Otu and Sayood (2003), and Tang et al. (2014). The accession numbers, species, and species name are listed in Table 1. All sequences were downloaded from NCBI GenBank.

**TABLE 1:** Names, species, and accession numbers for mitochondrial genomes of 30 mammalian species.

No	Accession no	Species	Sequence name
1	AJ002189	*Sus scrofa*	Pig
2	AJ010957	*Homo sapiens*	*Hippopotamus*
3	AJ001588	*Pan troglodytes*	Rabbit
4	U96639	*Canis familiaris*	Dog
5	AF010406	*Ovis aries*	Sheep
6	V00662	*Homo sapiens*	Human
7	U20753	*Felis catus*	Cat
8	X72004	*Halichoerus grypus*	Gray seal
9	D38115	*Pongo pygmaeus*	Orangutan
10	V00654	*Bos taurus*	Cow
11	X97337	*Equus asinus*	Donkey
12	D38116	*Pan troglodytes*	Common chimpanzee
13	D38113	*Pan paniscus*	Pigmy chimpanzee
14	Z29573	*Didelphis virginiana*	Opossum
15	Y10524	*Macropus robustus*	Wallaroo
16	X99256	*Hylobates lar*	Gibbon
17	Y18001	*Papio hamadryas*	Baboon
18	X97336	*Rhinoceros unicornis*	Indian rhinoceros
19	Y07726	*Ceratotherium simum*	White rhinoceros
20	X63726	*Phoca vitulina*	Harbor seal
21	AJ238588	*Sciurus vulgaris*	Squirrel
22	AJ001562	*Glis glis*	Fat dormouse
23	AJ222767	*Cavia porcellus*	Guinea pig
24	X79547	*Equus caballus*	Horse
25	X14848	*Rattus norvegicus*	Rat
26	V00711	*Mus musculus*	Mouse
27	D38114	*Gorilla gorilla*	*Gorilla*
28	X61145	*Balenoptera physalus*	Fin whale
29	X72204	*Balenoptera musculus*	Blue whale
30	X83427	*Ornithorhyncus anatinus*	Platypus

#### Dataset 2

The second dataset for analysis is the HIV-1 dataset studied in Ma et al. (2020). This dataset contains 43 HIV genome sequences used in Wu et al. (2007) and a controversial taxonomic sequence used in Chang et al. (2014). The dataset includes subtypes A, B, C, D, F, G, J, K, and H of the HIV-1 M, O, N groups and the CPZ sequence. The area, accession numbers, and subtypes are listed in Table 2. All these sequences were downloaded from NCBI GenBank.

**TABLE 2:** Accession numbers, subtype, and area for 44 HIV-1.

No	Area	Accession no	Subtype
1	Belgium (DRC)	AF084936	G
2	Finland (Kenya)	AF061641	G
3	Sweden (DRC)	AF061642	G
4	Belgium	AF190128	H
5	Belgium	AF190127	H
6	Cent. Afr. Rep	AF005496	H
7	Tanzania	AF447763	CPZ
8	Cameroon	L20571	O
9	Senegal	AJ302647	O
10	Cameroon	L20587	O
11	Cameroon	AY169812	O
12	India	AF067155	C
13	South Africa	AY772699	C
14	Ethiopia	U46016	C
15	Brazil	U52953	C
16	Cameroon	AY371157	D
17	DRC	K03454	D
18	Uganda	U88824	D
19	Somalia	AF069670	A1
20	Uganda	AF484509	A1
21	Uganda	U51190	A1
22	Kenya	AF004885	A1
23	DRC	AF286238	A2
24	Cyprus	AF286237	A2
25	Sweden	AF082395	J
26	Sweden	AF082394	J
27	Cameroon	AJ249239	K
28	DRC	AJ249235	K
29	Cameroon	AJ249237	F2
30	Cameroon	AY371158	F2
31	Cameroon	AJ249236	F2
32	Cameroon	AF377956	F2
33	Finland	AF075703	F1
34	France	AJ249238	F1
35	Brazil	AF005494	F1
36	Belgium (DRC)	AF077336	F1
37	Cameroon	AJ271370	N
38	Cameroon	AY532635	N
39	Cameroon	AJ006022	N
40	Netherlands	AY423387	B
41	Thailand	AY173951	B
42	Australia	Gray seal	B
43	France	K03455	B
44	U.S.	AY331295	B

We use two approaches to validate the method. First, we use the Robinson-Foulds (RF) distance to compare our method with other alignment-free methods. Second, we use the bootstrap method to construct consensus trees and show the stability of the trees obtained by our method.

## Methods

Let *S* = $s_{1}s_{2}\cdots s_{L}$ be a DNA sequence with length *L*, $a_{1}a_{2}\cdots a_{k}$ is a *k*-mer, where $a_{i}$ ∈(A,T,C,G). If the *k*-mer $a_{1}a_{2}\cdots a_{k}$ occurs in *S*, we denote by $p_{a_{1}a_{2}\cdots a_{k}}$ the vector composed of the positions of $a_{1}a_{2}\cdots a_{k}$ in this given sequence and by $p_{a_{1}a_{2}\cdots a_{k}\;}\left(i\right)$ its *i*th element. If the *k*-mer $a_{1}a_{2}\cdots a_{k}$ does not occur in *S*, $\text{ we set }p_{a_{1}a_{2}\cdots a_{k}}$ =(0). For example, for the DNA sequence GTA​ACC​TGA​ACG​TAC​TTG​GA with length 20, we list all 2-mer position vectors:

*P*_*AA*_=(3,9); *P*_*AC*_=(4,10,14); *P*_*AG*_= (0); *P*_*AT*_= (0); *P*_*CA*_=(0); *P*_*CC*_=(5); *P*_*CG*_=(11); *P*_*CT*_=(6,15); *P*_*GA*_=(8,19); *P*_*GC*_=(0); *P*_*GG*_=(18); *P*_*GT*_=(1,12); *P*_*TA*_=(2,13); *P*_*TC*_ = 0; *P*_*TG*_=(7,17); *P*_*TT*_=(16).

In this example, the 2-mers AG, AT, CA, GC, and TC do not appear. For each *k*-mer, its position vector provides its position distribution information in the sequence. One can use the *k*-mer position vectors to reconstruct the DNA sequence (Ma et al., 2020).

Ma et al. (2020) defined the position-weighted measure $D\left(a_{1}a_{2}\cdots a_{k}\right)$ of $a_{1}a_{2}\cdots a_{k}$ based on its position in the sequence as

$$D\left(a_{1}a_{2}\cdots a_{k}\right)=\{\begin{array}{cc} \frac{\sum_{i=1}^{n}p_{a_{1}a_{2}\cdots a_{k}}\left(i\right)}{L\left(L-k+1\right)}, & n\neq 0,\\ 0, & n=0, \end{array}$$ (1)

where *n* is the length of the vector $p_{a_{1}a_{2}\cdots a_{k}}$ . Actually $p_{a_{1}a_{2}\cdots a_{k}\;}\left(i\right)/L$ means the position weight of $a_{1}a_{2}\cdots a_{k}$ in the given sequence with length *L*.

We denote by *N* the number of sequences in a dataset. In order to characterize the importance of *k*-mers in the whole dataset, we count the number *m* of the sequences that contain a *k*-mer $a_{1}a_{2}\cdots a_{k}$ . Then the occurrence frequency *F* $\left(a_{1}a_{2}\cdots a_{k}\right)$ of this *k*-mer in the whole dataset is defined as *m*/*N*. We introduce the Shannon entropy *H*( $a_{1}a_{2}\cdots a_{k}$ ) of frequency *F*( $a_{1}a_{2}\cdots a_{k}$ ) defined by Murray et al. (2017) as

$$H\left(a_{1}a_{2}\cdots a_{k}\right)=-\left(F\log_{2}\left(F\right)+\left(1-F\right)\log_{2}\left(1-F\right)\right),$$ (2)

where *F* stands for *F* ( $a_{1}a_{2}\cdots a_{k}$ ).

In this study, we aim to get more DNA phylogenetic information by combining the above two methods and defining

$$E\left(a_{1}a_{2}\cdots a_{k}\right)=D\left(a_{1}a_{2}\cdots a_{k}\right)\times H\left(a_{1}a_{2}\cdots a_{k}\right)$$ (3)

Here, we regard Shannon entropy *H* ( $a_{1}a_{2}\cdots a_{k}$ ) as another weight.

For a fixed *K*, there are 4^*K*^ *k*-mers. For each *k*-mer $a_{1}a_{2}\cdots a_{k}$ , we can calculate the corresponding $E\left(a_{1}a_{2}\cdots a_{k}\right)$ , then arrange 4^*K*^ of these $E\left(a_{1}a_{2}\cdots a_{k}\right)$ to get a feature representation vector ( $E_{1},E_{2},\cdots ,E_{4^{K}}$ ) according to the alphabet order of the 4^*K*^ *k*-mers for each genome.

For two given genome sequences *A* and *B*, we can obtain $\;E_{\text{A}}$ = $\left(E_{1}^{A},E_{2}^{A},\cdots ,E_{4^{K}}^{A}\right)$ and $E_{\text{B}}=\left(E_{1}^{B},E_{2}^{B},\cdots ,E_{4^{K}}^{B}\right)$ by the method. We use the Manhattan distance to calculate the pairwise distance between these two genome sequences:

$$D\left(A,B\right)=\sum_{i}^{4^{K}}\left|\left(E_{i}^{A}-E_{i}^{B}\right)\right|$$. (4)

For a given dataset, we can derive a distance matrix by Eq. 4. This distance matrix contains the sequence similarity information. After obtaining the distance matrix, we insert it into the mega 7.0 software (Sudhir et al., 2016) and use Neighbor-Joining (NJ) program (Saitou et al*.* 1987) to construct the phylogenetic tree.

### Robinson-Foulds Distance and the Bootstrap Method

We use the Robinson-Foulds (RF) distance (Robinson and Foulds 1981) to judge the quality of the method. A smaller RF value means a closer distance between the phylogenetic tree and the reference tree.

(Yu et al., 2010) proposed a modified version of the bootstrap method to evaluate the reliability of the constructed phylogenetic tree. We also use this method in the present work. Its workflow is as follows: Each row is the feature vector ( $E_{1},E_{2},\cdots ,E_{4^{K}})$ of a species, and each column is the feature value of all genome sequences based on the same *k*-mer. Through random sampling of all columns, in which some columns may be selected many times, while some columns may not be selected at all, we randomly select one column. After 4^*K*^ times of selection, a new *N* × 4^K^ feature matrix is constructed. Using the new feature matrix, the Manhattan distance of any two rows is calculated to get a new distance matrix. Then we use the NJ method to construct a phylogenetic tree and repeat the above steps 100 times. Finally, a consensus tree is drawn by using consense. exe in the Phylip package. The frequency of a particular branch of a phylogenetic tree can be used as a measure of the stability of this branch.

## Results

### Experiment 1

**FIGURE 1:**
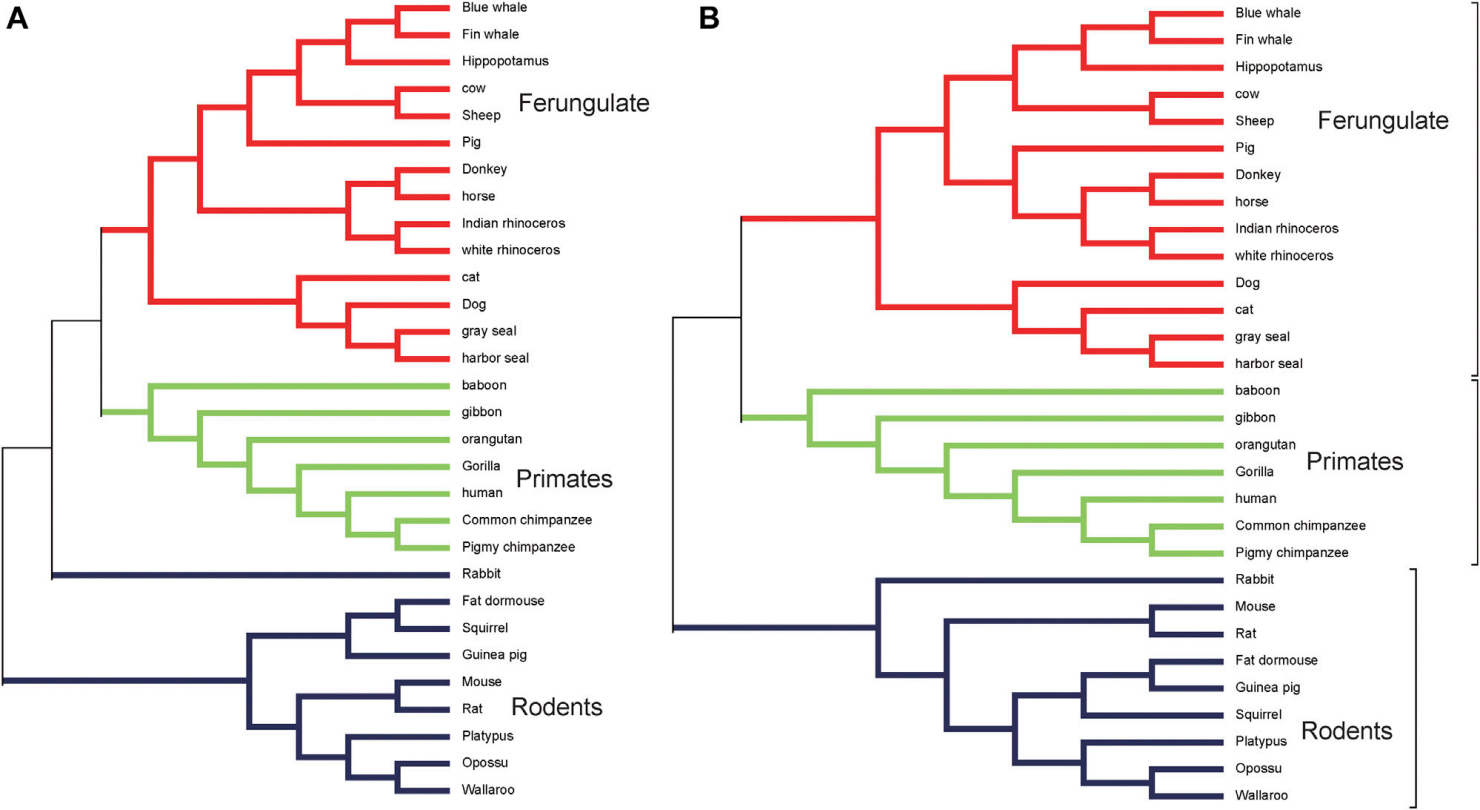
**(A)** The phylogenetic tree of 30 mammalian species reconstructed by ClustalX. **(B)** The phylogenetic tree of 30 mammalian species at *K* = 8 based on our method.

We use the genomes of 30 mammalian species in dataset 1 to construct a phylogenetic tree using ClustalX (Larkin et al*.* 2007) as the reference tree. ClustalX is one of the widely used multiple alignment programs. The result is shown in Figure 1A. It is seen that rabbit, fat dormouse, squirrel, guinea pig, mouse, rat, platypus, opossum, and wallaroo belong to the rodents group; human, baboon, orangutan, gibbon, gorilla, pigmy chimpanzee, and common chimpanzee belong to the primates group; blue whale, fin whale, hippopotamus, cow, sheep, pig, donkey, horse, Indian-rhinoceros, white rhinoceros, cat, dog, gray seal, and harbor seal belong to the ferungulates group. When *K* < 5, it is not feasible to construct a phylogenetic tree using our method. When *K* = 5, 6, the 30 mammals cannot be divided into three groups in our tree. When *K* = 7, it can be divided into three groups, but the relationship between guinea pig and fat dormouse is not correct. When *K* = 8, 9, the branches of the tree become correct. We list the RF distances between the phylogenetic tree constructed by our method at *K* = 5, 6, 7, 8, 9 and the reference tree constructed by ClustalX in Table 3. From Table 3, we can see that the RF distance reaches the minimum when *K* = 8. We show the phylogenetic tree of *K* = 8 constructed by our method in Figure 1B. From Figure 1B, we can see that the species in the three main categories are grouped correctly. Primates and ferungulates are closer, and this relationship is consistent with that in Figure 1A. In terms of branches, monotremes (platypus), marsupials (wallaroo, opossum), murid rodents (mouse, rat), non-murid rodents (guinea pig, squirrel, fat dormouse, rabbit), perissodactyls (white rhinoceros, horse, Indian rhinoceros, donkey), carnivores (harbor seal, dog, gray seal, cat), artiodactyls (sheep, cow, hippopotamus, pig), primates (human, pigmy chimpanzee, common chimpanzee, gorilla, baboon, gibbon, orangutan), and cetaceans (blue whale, fin whale) are grouped into respective taxonomic classes accurately.

**TABLE 3:** The RF distance between the phylogenetic tree conducted by our method at K = 5,6,7,8,9 and the reference tree conducted by ClustalX.

*K*	5	6	7	8	9
RF distance	38	28	22	8	10

**FIGURE 2:**
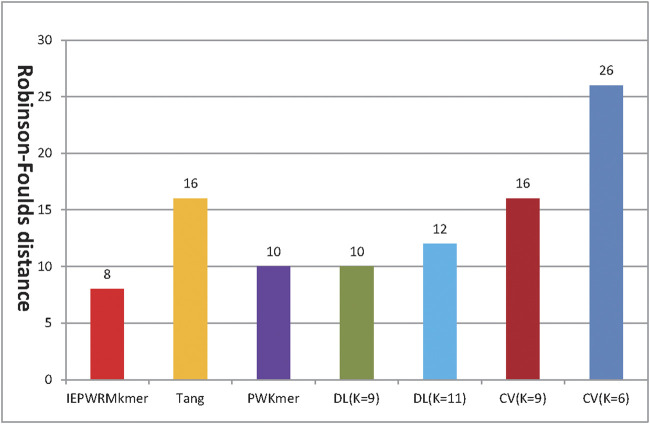
The Robinson–Foulds distance between the tree reconstructed by ClustalX method and the phylogenetic trees reconstructed by our method (IEPWRMkmer K = 8), the CVTree method, the DLTree method, Tang’s method (K = 7), and the PWKmer method (K = 9) on dataset 1 (we used the optimal tree by CVTree and DLTree).

Figure 2 shows the RF distance between the reference tree constructed by ClustalX and the phylogenetic tree constructed by our method, Tang’s method, PWKmer, DLtree, and CVtree on dataset 1. Using our method, when *K* = 8, the RF distance is 8. The shortest RF distance of DLtree (*K* = 9) is 10, the shortest distance of CVtree (*K* = 9) is 16, the shortest distance of Tang’s method (*K* = 7) is 16, and the shortest distance of *PWKmer* (*K* = 9) is 10. Therefore, the results of our method are closer to those of ClustalX than those of the other methods, which indicates that our method is effective.

**FIGURE 3:**
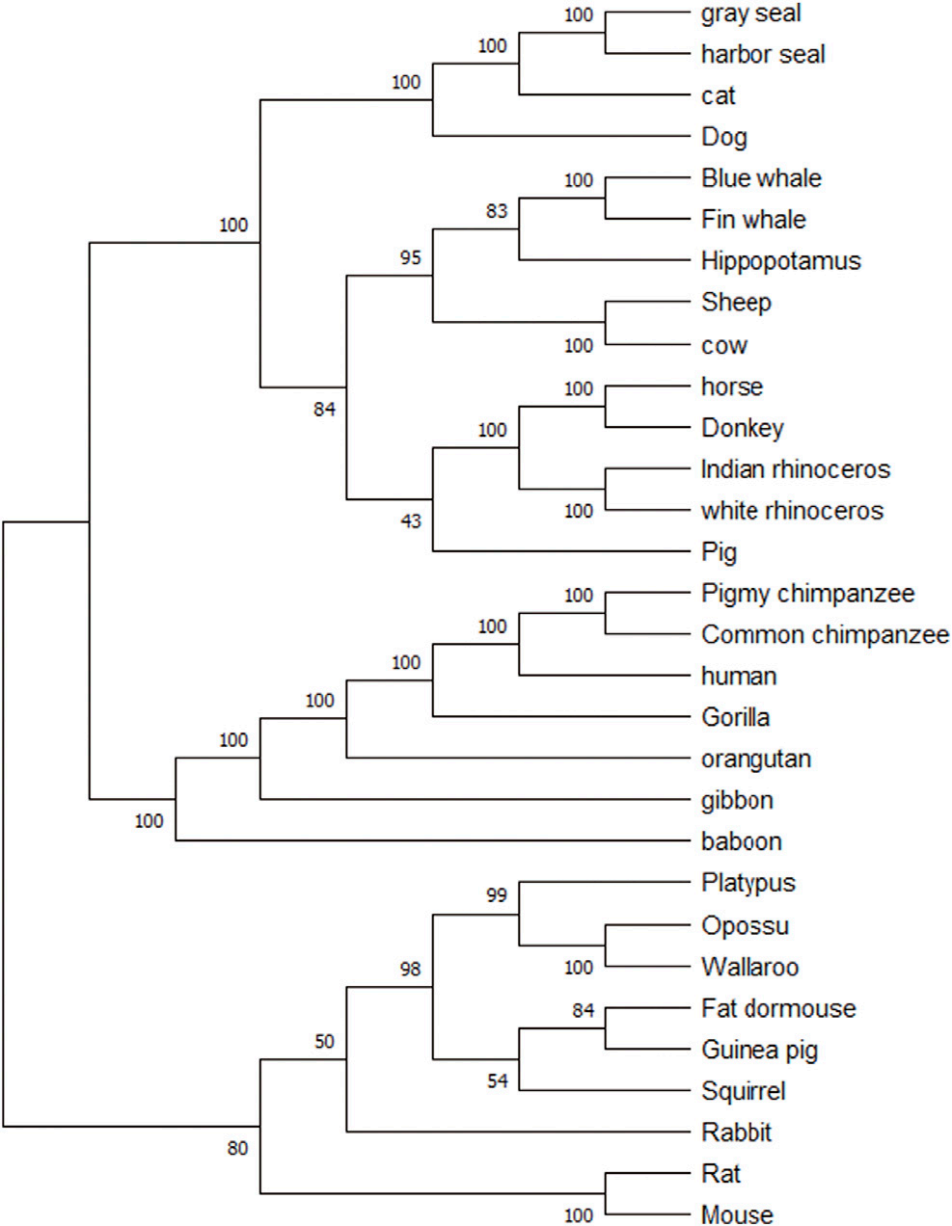
The modified bootstrap consensus tree for Figure 1B based on 100 replicates.

Figure 3 shows the consensus tree of 30 mammalian species based on our method. Compared with Figure 1B, 30 mammalian species are divided into the rodents group, the ferungulates group, and the primates group correctly. The support rate is 80% for the rodents group and 100% for both ferungulates and primates groups. Among the branches, marsupials (opossum, wallaroo), carnivores (dog, cat, harbor seal, gray seal), murid roots (rat, mouse), and cetaceans (fin whale, blue whale) are all supported by a 100% rate. In the artiodactyls group (cow, sheep, pig, hippopotamus), pig is separated out of the artiodactyls group, but the support rate is low at 43%. It indicates that the phylogenetic tree constructed by our method is quite robust.

### Experiment 2

The human immunodeficiency viruses (HIV) represent a group of retroviruses, which are not presumed to have originated from human cellular DNA sequences, hence are distinct from endogenous retroviruses (Wu et al., 2007). HIV-1 can be classified into three major phylogenetic groups, namely M (major), N (new), and O (others). Group M is responsible for the HIV pandemic, it is divided into nine subtypes, namely A, B, C, D, F, G, J, K, and H. Based on differential phylogenetic clustering, the subtypes A and F are further divided into sub-subtypes (A1, A2) and (F1, F2), respectively. Groups N and O are derived from other primates and then infect humans. CPZ is a non-human primate virus isolated from chimpanzees, which is closest to human-to-human transmission of HIV.

**FIGURE 4:**
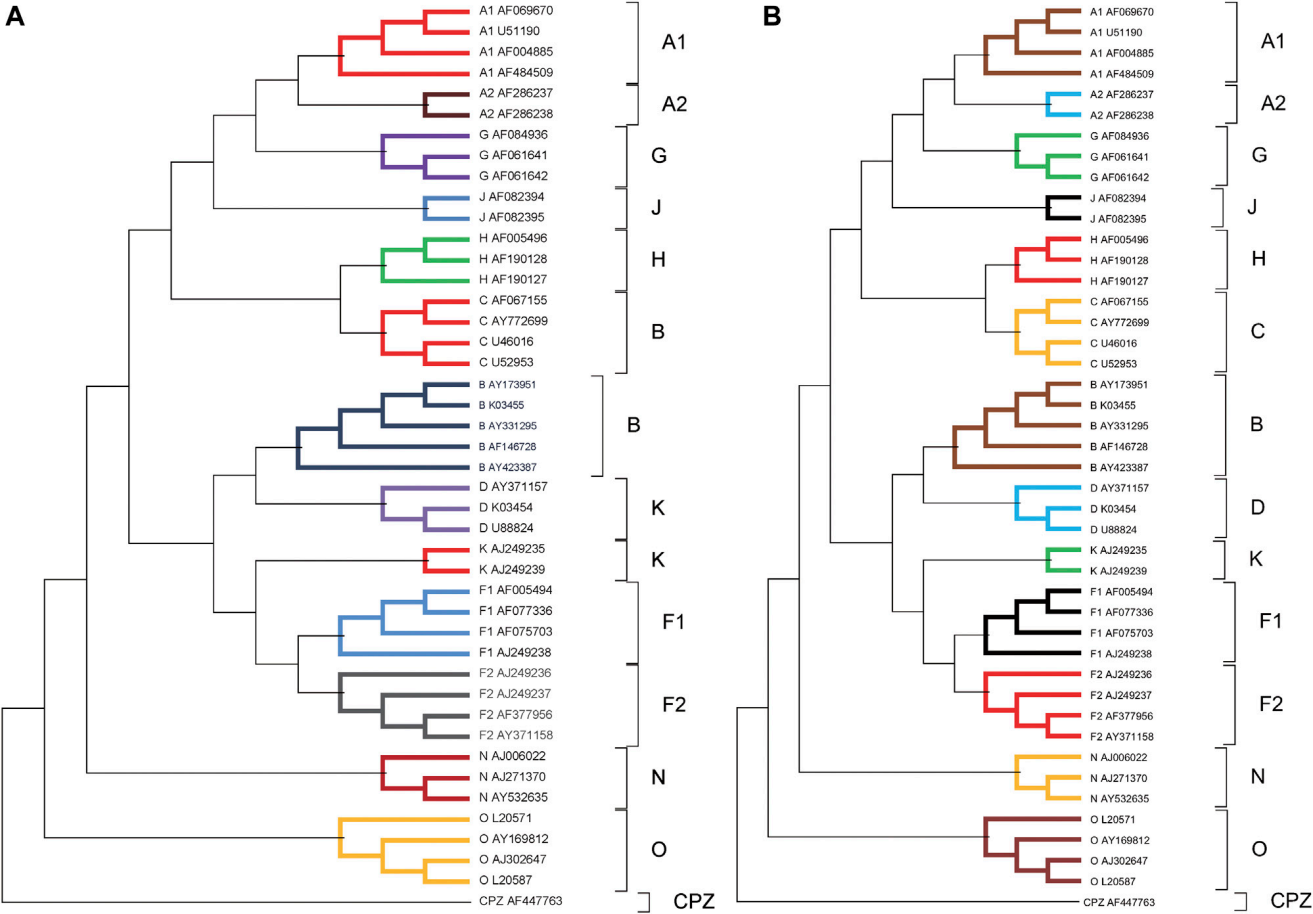
**(A)** The phylogenetic tree of 44 HIV-1 genomes reconstructed by ClustalX. **(B)** The phylogenetic tree of 44 HIV-1 genomes reconstructed by our method (*K* = 7).

We performed the phylogenetic analysis of 44 HIV-1 complete genome sequences in dataset 2 using ClustalX and our method. The phylogenetic trees reconstructed by ClustalX and our method (*K* = 7) are shown in Figure 4A and Figure 4B, respectively. From Figure 4B, we can see that the species from all subtypes can be correctly classified into their groups (A, B, C, D, F, G, J, K, H, O, and M), and CPZ as the reference sequence is separated into the outermost. From the internal branches, both F and A contain two subtypes (F1 and F2) and (A1 and A2), respectively. Our method can separate the two subtypes, and in the branches, both F and A subtypes can be closely grouped together.

**FIGURE 5:**
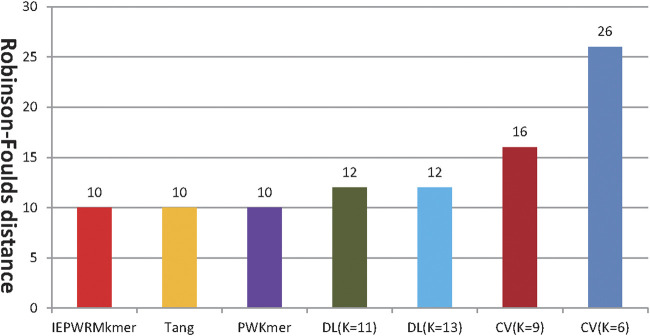
The RF distance between the reference tree constructed by Clustalx and the phylogenetic trees constructed by our method (IEPWRMkmer, *K* = 7), Tang’s method (*K* = 8), the PWKmer method (*K* = 9), the DLtree method, and the CVtree method. (For the PWKmer method, the DLtree method, and the CVtree method, we chose their optimal classification tree).

Figure 5 shows the RF distances between the reference tree constructed by ClustalX and the phylogenetic trees constructed by our method, Tang’s method, PWKmer, DLtree, and CVtree. Using our method, when *K =* 7, the RF distance is 10. The shortest RF distance of the DLtree (*K =* 11) is 12, the shortest distance of the CVtree (*K =* 9) is 16, the shortest distance of the PWKmer (*K =*9) is 10, and the shortest distance of Tang’s method (*K =* 9) is 10. Therefore, our method performs better than the DLtree and the CVtree on dataset 2 and has the same performance as Tang’s method and PWKmer. The results indicate that our method is quite effective again.

**FIGURE 6:**
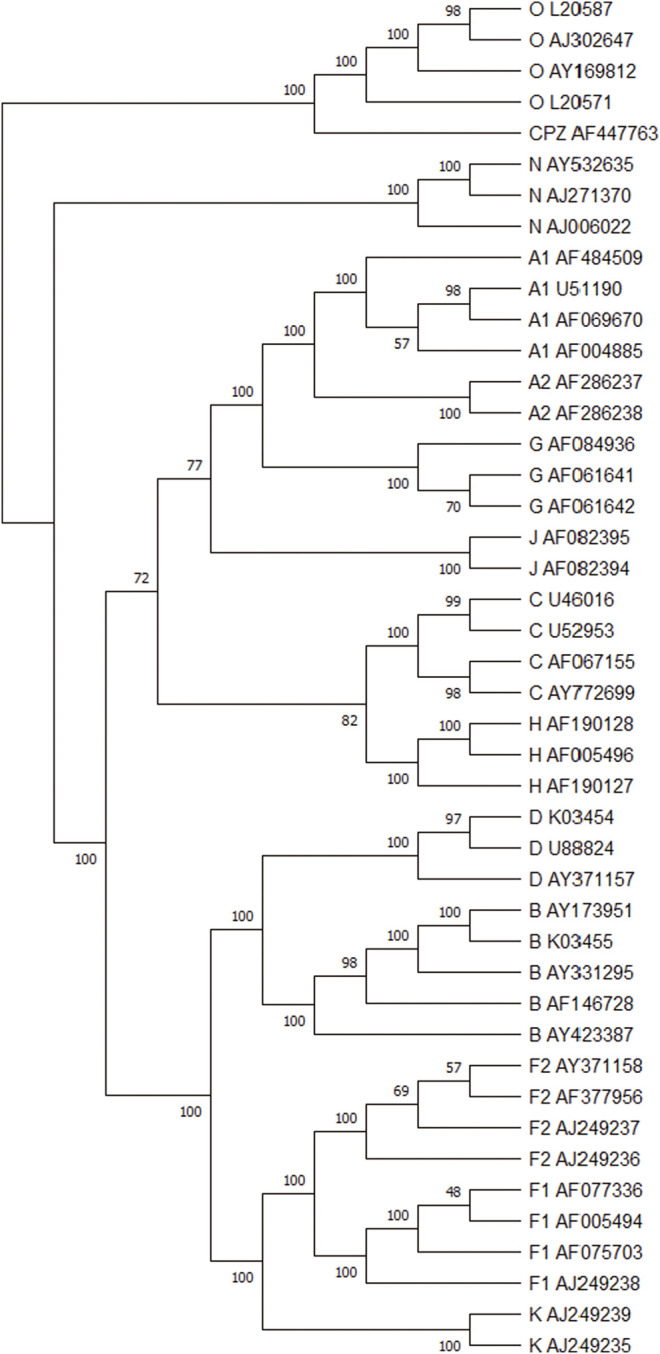
The modified bootstrap consensus tree for Figure 4B based on 100 replicates.

Figure 6 shows the consensus tree of 44 HIV-1 based on our method. Comparing with Figure 4B, all HIV-1 sequences are divided into the M, N, O, and CPZ groups, whose support rate is 100%. From the branch point of view, in group M, the branch support rate of all subtypes is 100%. For subtypes A and F, the subtypes (A1, A2) and (F1 and F2) are clustered with 100% support. It again indicates that the phylogenetic tree constructed by our method is quite robust.

### Estimate of the Optimal Parameter *K*

Different lengths of *k*-mers contain different phylogenetic information. Short *k*-mers may not contain sufficient DNA sequence information. Long *k*-mers contain sufficient phylogenetic information, but it needs large memory and takes a long time to calculate the distance based on information on long *k*-mers. Therefore, it is also very important to estimate an optimal value of *K* as heralded in (Yu et al., 2010) for the DLTree method and (Qi et al., 2004) for the CVTree method.

In this paper, we propose to use the Shannon entropy of the feature matrix to determine the optimal value of *K*. Using Eq. 3, we can obtain an *N* × 4^*K*^ feature matrix for a dataset with *N* genomes. Then, we propose to define a scoring strategy as

$$\text{score}\left(K\right)=-\frac{1}{N}\sum_{j=1}^{N}\sum_{i=1}^{4^{K}}\left(E_{ij}\log_{2}E_{ij}+\left(1-E_{ij}\right)\log_{2}\left(1-E_{ij}\right)\right).$$ (5)

The optimal *K* is the value at which score(K) reaches its maximum.

**FIGURE 7:**
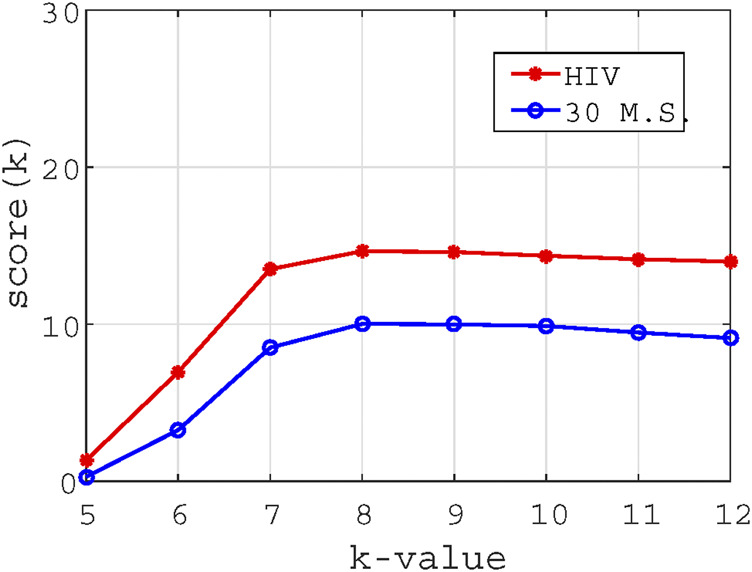
The trend chart of *K* value vs scoring measure score(K) . The red circles represent the scores of the dataset of 30 mammalian species for different *K* values, and the blue dots represent the scores of the HIV dataset for different *K* values.

We use Eq. 5 to calculate score(K) on datasets 1 and 2 for different *K*. The relationship between score(K) and *K* is shown in Figure 7 for these two datasets. It is seen that score(K) reaches the largest value when *K* = 8 on the two datasets. Considering that the larger *K* is, the more memory resources are consumed, we only consider the values near *K* = 8 (e.g., *K* = 7, 8, 9). For the 30 mammalian species dataset, we have seen that the phylogenetic tree for *K* = 8 constructed by our method is closest to the reference tree. The same happened for the HIV-1 dataset with *K* = 7. The outcomes indicate that score(K) can provide an effective means to estimate the optimal value of *K*.

## Conclusion

In this paper, a new alignment-free method is proposed for phylogenetic analysis and sequence comparison based on whole genome sequences. Our method combines the position-weighted measure of *k*-mers and the information entropy of frequency of *k*-mers. We used the Manhattan metric to measure the distance between a pair of sequences and the NJ method to construct the phylogenetic tree. In order to test the effectiveness and reliability of our method, we applied it on two datasets of 30 mammalian species and 44 HIV-1 genomes. The results demonstrated that the present method is efficient and reliable. A suitable *K* value is important to capture rich phylogenetic information of DNA sequences. In order to choose an optimal *K* value, we proposed a scoring measure based on the information entropy. The obtained results on two real datasets support that the method can capture the *k*-mer distribution information and is effective for whole genome sequence comparison and phylogenetic analysis.

Remark: The method of this paper is derived from the two studies Ma et al. (2020) and Murray et al*.* (2017). There are differences between this work and previous works: Tang et al. presented the average relative distance for normalized *k*-mers. PWKmer uses the counts and position distributions of *k*-mers to capture more evolutionary information. KWIP (Murray et al*.* 2017) uses information entropy to weight the inner product (Si ∗ Sj), while we use information entropy to weight the relative positions of *k*-mers. KWIP uses a kernel function to calculate the distance, while we use the Manhattan metric to calculate the pairwise distance between species. Here, we claimed that the results obtained by the IEPWRMkmer method are close to those by ClustalX and the IEPWRMkmer is superior to the other distance metrics. We used the phylogenetic tree constructed by ClustalX as the reference tree or standard tree, hence we cannot claim that our method is superior to the ClustalX method.

## Data Availability

The genome datasets analyzed for this study can be found in the GenBank https://www.ncbi.nlm.nih.gov/
